# 

**Published:** 1914-10-24

**Authors:** 


### Medical Appointments.

Mr. G. Paterson Burr, M.B., Ch.B. Aberdeen, has been appointed to the post of house physician at Bradford Royal Infirmary.

Mr. Georg Herman Monrad Krohn, sixth assistant medical officer at Cane Hill Mental Hospital, has been promoted to be fourth assistant medical officer.

Dr. H. A. Cookson has been appointed house surgeon at the Torbay Hospital, Torquay, in place of Dr. S. J. A. H. Walshe, who is now with the troops in France.

Mr. R. G. J. McEntire, fifth assistant medical officer at Cane Hill Mental Hospital, has been promoted to be fourth assistant, and Mr. Allen Coulter Hancock has been appointed to be fifth assistant medical officer.

Mr. J. C. Wooton, second assistant medical officer at Cane Hill Mental Hospital, having resigned his appointment, Mr. Frederick Morres, the third assistant, has been promoted to fill the vacancy.

Mr. Chas. H. Budd, M.B., Ch.B. Oxon, late house physician at Addenbrooke's Hospital, Cambridge, and who is now in charge of Mr. Chas. F. Searle's practice at Cambridge, has been appointed honorary anæsthetist at Addenbrooke's Hospital, Cambridge.

Dr. Charlotte M. Birnie, M.B., Ch.B., has been appointed junior assistant medical officer at Camberwell Infirmary. She has been acting as *locum tenens* at the infirmary, but was previously junior assistant medical officer at The Retreat, York, from March 1913 to October 1914; resident medical officer at the Jewish Hospital, Manchester, from October 1912 to March 1913; and resident house surgeon at the Elder Cottage Hospital, Govan, from November 1911 to May 1912.

With the approval of the Chairman of the London County Council, Mr. C. Luxmoore Drew, Coroner for the Western District, has appointed Mr. Peter Byrne as his deputy in the place of Mr. Alfred Douglas Cowburn, who has resigned on taking service with the Fleet.

### Buildings Contemplated.

Lochwinnoch.—The Paisley Town Council have approved plans prepared by the Joint Sanatorium Board of the County of Renfrew for a sanatorium and farm colony at West Michelton and Peockstone, Lochwinnoch. The estimated cost, after deducting £18,850 to be contributed by the Renfrewshire King Edward Memorial Committee, and a grant of £8,850 from the Local Government Board, will be £23,811.

Manor Mental Hospital.—It is proposed to build an additional two-storey block at Manor Mental Hospital, which will provide quarters for the assistant matron and separate rooms for seventeen nurses.

Morpeth.—The Guardians have resolved to obtain plans and estimates for a new infirmary, to cost about £7,500.

Neath.—The Glamorgan County Council have decided to apply to the Local Government Board for sanction to borrow £14,000 for the Neath Joint Hospital.

### Gifts and Bequests.

Mr. George Frederick Hopkins, who has left estate of which £20,902 is net personalty, has, subject to certain legacies, left the ultimate residue of his estate to the Hospital for Diseases of the Throat, Nose, and Ear, Golden Square, W.

Mr. Robert Jackson Kent has left £500 each to the Free Cancer Hospital and the Middlesex Hospital, in each case to be applied towards the discovery of the cause of cancer, the means of prevention of its development, and of a cure therefor.

Miss Margaret Drysdale, of Crosshill, Glasgow, has left to the Royal, Western, and Victoria Infirmaries, Glasgow, £1,000 each; to the Royal Glasgow Asylum for the Blind, £250; to the Glasgow Samaritan Hospital for Women, £250; to the West of Scotland Convalescent Seaside Home, Dunoon, £200; to the Royal Hospital for Sick Children, Glasgow, £100; to the Ladies' Auxiliary of the Association for the Relief of Incurables in Glasgow and the West of Scotland, £200; to the Glasgow Hospital for Women, Elmbank Crescent, £100; to the Glasgow Hospital for Diseases of the Ear, £100; to the Glasgow Cancer Hospital, £100; to the Eastpark Home for Infirm Children, Glasgow, £100; to the Glasgow Institution for the Deaf and Dumb, £150; to the Cripple Children's League of the Glasgow United Evangelistic Association, £100. The testator also directed her trustees to distribute the residue of her estate among the charitable and benevolent institutions mentioned.

### A SOUVENIR OF "CANADA'S GIFT."

Canada is making a splendid gift of flour to the Mother Country. It has been decided that the sacks, when empty, should be sold as souvenirs at 5s. each. Two-thirds of this sum will be devoted to the Prince of Wales's National Relief Fund, and one-third to the Belgian Refugees' Fund. The sacks are all marked "Canada's Gift." Applications for the sacks as souvenirs, accompanied by a remittance of 5s., should be sent to the Hon. Secretaries, National Relief Fund, York House, St. James's Palace, London, S.W.

### The Book Market.

LIST OF NEW BOOKS RECEIVED FROM THE FOLLOWING PUBLISHERS:—

The Medical Publishing Co., Ltd.: "The Operative Treatment of Fractures." Second Edition. By Sir W. Arbuthnot Lane, Bart., M.S., F.R.C.S. 10s.

A. and C. Black: "Tuberculosis of the Bones and Joints in Children." By J. Fraser, M.D., F.R.C.S. 15s. net.

Cassell and Co.: "Elements of Surgical Diagnosis." Fourth Edition. By Sir Alfred Pearce Gould, K.C.V.O., M.S. 10s. 6d. net.

T. Werner Laurie, Ltd.: "A War Cookery Book for the Sick and Wounded." By Jessie M. Laurie. 6d. net.

Sampson Low, Marston and Co., Ltd.: "Low's Handbook to the Charities of London, 1914." 1s. net.

Constable and Co. (for the General Medical Council): "The British Pharmacopœia, 1914."

H. Kimpton: "Life: Its Origin, and Energy Mechanism." By Jadroo. 1s. net. "Nutrition, a Guide to Food and Dieting." By Chas. E. Sohn, F.I.C., F.C.S. 3s. 6d. net.

Longmans and Co.: "Essentials of Physiology." By F. A. Bainbridge, M.A., M.D., and J. Acworth Menzies, M.D. 10s. 6d. net.

Methuen and Co.: "Nursing in War Time." By M. N. Oxford. 1s. net.

Oxford University Press: "Bacilli and Bullets." By Sir Wm. Osler, Oxford Pamphlets, 1914. 1d. net.

Scientific Press, Limited: "Elementary Bacteriology for Nurses." By G. Norman Meachen, M.D. 2s. net. "First Lines in Nursing." By E. Margaret Fox. 2s. 6d. net.

University Tutorial Press, Limited: "The London Matriculation Directory." No. 68. 1s. net.

### Pamphlets and Papers.

"Cancer and the Doctors: Humanity Betrayed." By John Shaw, M.D. London. Price (post free) 3d. Published by the Author, Mühlehorn, Switzerland.

"What is Malaria and the Germ Theory?" Translated from the Bengali of Vaidyaratna K. K. Vidyabhushan.

"The Economic Progress of the United States during the last Seventy-five Years." By Frederick L. Hoffman, LL.D.

"Tasty Ways of Cooking Fish," with an Introduction on the Good Value of Fish. By Sir James Crichton Browne, M.D. Issued by the National Sea Fisheries Protection Association, Fishmongers' Hall, E.C.

### Institutional and Public Reports.

King Edward's Hospital Fund for London: Statistical Report on the Expenditure of One Hundred and Six London Hospitals for the Year 1913.

Brighton County Borough Mental Hospital, Hayward's Heath: Tenth Annual Report.

Royal Victoria Hospital, Dundee: Fourteenth Annual Report.

Borough of Portsmouth: Report on the Municipal Disinfectant Station of Electrolysed Sea-water Disinfectant. By A. Mearns Fraser, M.D., M.O.H.

County Borough of Halifax Health Department: Annual Report for Fifty-two Weeks ended December 27, 1913. By James T. Leech, M.D., D.P.H.

Report of a Committee appointed by the Council of the Royal Institute of Public Health to consider the Standardisation of Methods for the Bacterioscopic Examination of Water.

Report upon the Work of the Tuberculin Dispensaries of Derbyshire during the Months of May 1913 and May 1914. By J. Court, M.R.C.S., L.R.C.P. [See The Hospital, September 12, p. 644.]

King Edward VII.'s Hospital, Cardiff: Seventy-seventh Annual Report for 1913.

Rates of Subscription: Twelve months, 6/6; Six months, 4/-; Three months, 2/-, post free to any address in the United Kingdom. Abroad, Twelve months, 8/8; Six months, 5/-; Three months, 2/6, post free.—Address The Subscription Dept., "The Hospital," The Hospital Building, 28/29 Southampton Street, Strand, London W.C.

